# Caregiving and mental well-being: the role of caregivers’ age and insomnia

**DOI:** 10.3389/fpsyt.2025.1482890

**Published:** 2025-04-29

**Authors:** Lawrence T. Lam

**Affiliations:** ^1^ Faculty of Medicine, Macau University of Science and Technology, Macau SAR, China; ^2^ Faculty of Medicine and Health, The University of Sydney, Sydney, NSW, Australia; ^3^ Faculty of Health, University of Technology Sydney, Sydney, NSW, Australia

**Keywords:** caregiving, mental well-being, insomnia, age effect, population survey

## Abstract

**Objective:**

While the impact of caregiving on physical and mental health has been studied, there remains a gap in understanding the relationship between caregiving and mental well-being within a robust theoretical framework. Moreover, existing research provides mixed findings regarding the burden experienced by caregivers and its impact on their health. To address this gap, we explore the association between caregiving and mental well-being, considering age as a potential moderator. Additionally, we examine the role of insomnia in this context.

**Methods and materials:**

This population-based cross-sectional survey recruited adult residents in Macau. Participants’ caregiving roles were assessed, and their mental well-being was measured using the Short Warwick Edinburgh Mental Wellbeing Scale (SWEMWBS). Insomnia was also evaluated as a potential moderator by Insomnia Severity Index (ISI). The moderating effects of age and insomnia in the relation between caregiving and mental well-being were examined, with adjustments made for confounding variables using regression analyses.

**Results:**

After controlling for confounders, the results indicated a significant interaction between caregiving roles and age group (β=0.84, s.e. = 0.10, t=8.42, p<0.001). Subgroup analyses revealed that the association between caregiving roles and mental well-being was significantly moderated by insomnia in the 18-34 age group (β=1.55, s.e. = 0.55, t=2.81, p=0.005), but not in other age groups. Younger caregivers seemed to benefit more from their caregiving roles in terms of mental well-being compared to older caregivers.

**Conclusions:**

The results highlight the importance of considering age and insomnia when examining the impact of caregiving on mental well-being. The findings suggest that being a caregiver may have positive effects on mental health, particularly among younger individuals. Further research is needed to explore the underlying mechanisms and develop targeted interventions for caregivers of different age groups.

## Introduction

The past decade has seen a rise in chronic diseases, leading to an increase in informal caregiving by family members. The World Health Organization defines caregivers as those providing formal and informal assistance to individuals with disabilities, long-term conditions, or the elderly, including emotional, financial, and hands-on help (1, 2). Despite the global trend, no comprehensive study has estimated caregiving prevalence. Data from the International Alliance of Carers Organisations (IACO) shows an estimated 260 million caregivers across 16 countries and 2 regions, with prevalence rates ranging from 4.5% in Taiwan to 21.3% in the USA (3).

Caregiving significantly impacts caregivers’ mental and physical health and economic well-being. Research shows caregiving is associated with psychological stress, anxiety, depression, physical health issues, and disruptions in social and family life (4–8). Financial strain is common among employed caregivers due to missed workdays, early retirement, and reduced working hours (9, 10). Caregivers often report fatigue, isolation, reduced quality of life, heightened burden, physical health issues, and suicidal thoughts (8, 11–16). Adolescents also experience emotional distress, physical health issues, academic setbacks, and psychosocial difficulties (17–23).

Mental well-being, synonymous with positive mental health, includes self-acceptance, personal growth, resilience, autonomy, and mastery of one’s environment (24). The World Health Organization emphasizes positive mental health for overall population well-being (25). Mental well-being has gained attention over the past two decades. WHO defines it as a state of well-being where individuals recognize their abilities, cope with normal stresses, contribute productively, and engage with their community (26). Measuring mental well-being requires thoughtful consideration (24). Instruments like the WHO-5, MHC-SF, and WEMWBS assess mental well-being. Tennant et al. introduced a two-dimensional model comprising hedonic and eudaimonic aspects (27, 28).

Recent research focuses on factors affecting mental well-being, particularly among adolescents (29). Sleep quality is crucial for mental well-being (30, 31). An Australian study found healthy sleep correlated with better mental well-being (31). However, studies specifically examining the link between sleep problems and mental well-being based on the WEMWBS model are lacking.

Caregiving research often emphasizes the burden and negative effects on caregivers, but positive aspects, especially among young caregivers, receive less attention. Caregiving can enhance young caregivers’ self-identity, self-worth, and sense of belonging (32). Adolescents involved in caregiving report increased self-esteem and interpersonal competence (33). The impact of caregiving on mental well-being appears age-dependent.

Current research on the relationship between caregiving and mental well-being remains limited. This study aims to explore the connection between caregiving and mental well-being, focusing on caregivers’ age and insomnia. It is hypothesized that caregivers’ age moderates the relationship between caregiving and mental well-being, and this relationship is further attenuated by insomnia. By investigating these factors, we aim to contribute valuable insights and inform targeted interventions for caregivers across different age brackets.

## Materials and methods

### Study design and participants

This was a population-based cross-sectional health survey using a self-reported online questionnaire for data collection. The survey was conducted between April and July 2024 and targeted adult residents living in Macau during the study period. All Macau residents who fulfilled the following requirements were eligible to participate in the survey: 1) Macau residents aged 18 years or above; 2) Able to read Chinese since the survey questionnaire was presented in the Chinese language; 3) Individuals who migrated to Macau and living in Macau at the time of the survey. Macau residents who could understand and speak the Chinese language but could not comprehend written Chinese and individuals who migrated to Macau but were not living in Macau at the time of the survey were excluded from the study. Participants were recruited through the collaborating community and professional associations and societies. In total, 23 associations and societies were involved in the survey as collaborating partners. These associations and societies represented a wide range of industries and different sectors of the Macau population. The senior management of these associations and societies agreed to assist in distributing the online questionnaire to their members through internal communication channels. Members were encouraged to participate in the survey through public appeals and personal invitations. The total number of potential participants was more than 50,000, accounting for nearly 9% of the Macau residential adult population. This should be a sufficiently large sample frame for generating a representative sample. Ethics approval was granted by the Faculty Ethics Research Committee of the Faculty of Medicine, Macau University of Science and Technology (MUST-FMD-200402025001).

### Outcomes and exposure measures

The exposure variable, namely the caregiving role, was measured by a direct question asking whether the respondent has been caring for a family member or a friend without payment and the relationship with the person. Other main study variables were the respondent’s age and insomnia. The primary outcome of the study was the respondents’ mental well-being. The mental well-being and insomnia were assessed using validated instruments with the following details.

#### Mental Wel-being

Mental Well-being Participants’ mental well-being was assessed using the Warwick-Edinburgh Mental Well-being Scale (27). Tennant et al. proposed a two-dimensional model of mental well-being consisting of hedonic (subjective happiness and life satisfaction) and eudaimonic (psychological functioning and actualisation of potential) aspects (27). The WEMWBS was validated and widely used in many studies, showing good content and structural validity, high reliability (Cronbach’s alpha 0.89-0.91), and strong correlations with other mental health scales (27). This survey used the short form of the Short Warwick Edinburgh Mental Wellbeing Scale (SWEMWBS) (SWEMWBS ^©^ NHS Health Scotland, University of Warwick and University of Edinburgh, 2008, all rights reserved). The short scale was fully validated with ample information on its psychometric properties on the official website (([https://www.corc.uk.net/outcome-experience-measures/short-warwick-edinburgh-mental-wellbeing-scale-swemwbs/](https://www.corc.uk.net/outcome-experience-measures/short-warwick-edinburgh-mental-wellbeing-scale-swemwbs/)). The scale was translated and validated with good reliability of a Cronbach’s alpha of 0.89, a test-retest reliability of 0.68, and the item-total correlations ranging from 0.57 to 0.75 (16). For the construct validity, a single component was identified with an eigenvalue of 4.28 and 61.1% variance explained (16 **).** Raw scores were converted to matric scores and used as a continuous variable for data analysis.

#### Insomnia

Insomnia was assessed using the Insomnia Severity Index (ISI) (34). The scale consists of seven items rated on a 5-point Likert scale, covering sleep quality, severity of symptoms, satisfaction with sleep patterns, interference with daily functioning, noticeability to others, and overall distress caused by sleep problems. The ISI demonstrated good reliability and validity, with Cronbach’s alpha of 0.74 and item-total correlations ranging from 0.36 to 0.54 (34). The severity of insomnia was classified as 0-7 for no clinically significant insomnia, 8-14 for subthreshold insomnia, 15-21 for moderately severe insomnia, and 22-28 for severe insomnia. The Chinese version was validated with a Cronbach’s alpha of 0.81 and item-to-total correlations ranging between 0.34 and 0.67 (35). This study used a dichotomised grouping of moderate/severe and none/minimal/mild for ease of analysis.

Data were also collected on demographics, including age, sex, education level, and employment status. Information on potential confounding variables that might influence the relationship between caregiving role and mental well-being was also gathered. These included the number of stressful concerns, help-seeking intention, and coping with stress.

### Sample size and data analysis

The sample size was calculated based on the study design and the data analysis method. It was assumed that 20% of the Macau population did not achieve an optimal level of mental well-being. With a study power of 80% for detecting the true estimate at a type I error rate of 5%, a margin of error of 3%, and an attrition rate of 10%, a sample size of about 1200 was estimated.

Data were analysed using the STATA statistical software (StataNow 18.5). Descriptive statistics were generated for the outcome and exposure variables, as well as other study variables. The data were summarised for continuous variables using mean, standard deviation, median, and interquartile range. Frequency and percentage were used to report the summary of categorical variables. Since the outcome variable was continuous, bivariate associations between the exposure, other study variables, and the outcome variable were analysed using parametric tests, including Student’s t-test or Oneway ANOVA, to compare means between or among groups. Correlation was used to examine the unadjusted relationship between two continuous variables. Multiple regression modelling techniques were applied to analyse the relationship between exposure and outcome variables and explore the moderating effect of respondents’ age and insomnia after adjusting for potential confounding factors. A type I error rate of 5% was applied for all hypothesis testing.

## Results

In total, 1460 completed responses were collected through the online survey platform. Since the platform only provided data on the completed questionnaires, the response rate was unknown. The descriptive information on the demographics, the study and the outcome variables, as well as the other potential confounding variables, was summarised in **Table 1**. As shown, the majority of the respondents were females (n=1001, 68.6%) and younger, with nearly 45% in the age group of 18-34 years (n=655, 44.9%). Slightly less than half of the respondents were married or in a de facto relationship (n=693 47.5%), and two-thirds attained an education level of university or higher (n=966, 66.2%) and mostly were working full-time (n=925, 63.4%). In terms of the study variables, namely having a caring role and insomnia, 46% (n=672) of respondents reported that they were playing a caregiver’s role in offering care to someone. Among the caregivers, the most common role was caring for infants and young children, with nominations from 321 (22.0%) respondents. This was followed by caring for other unspecified people (n=237, 16.2%). Two hundred and seventy-nine (n= 279) (19.1%) respondents were classified as having moderate to severe insomnia. This represented 19.1% of the sample. For mental well-being, the sample mean was 21.5 (s.d. = 4.9) out of a maximum score of 35, with 21.2 (s.d. = 5.6) and 21.6 (s.d. = 4.6) for males and females, respectively.

**Table 1:** Descriptive information of the demographics and other variables, caring roles, insomnia, and mental well-being of the sample (N=1460).

Variables	Frequency (%) or Mean (s.d.)
Demographics	
Sex	
Male	459 (31.4%)
Female	1001 (68.6%)
Age group (years)	
18-34	655 (44.9%)
35-54	589 (40.3%)
55 +	216 (14.8%)
Marital status	
Married/De facto	693 (47.5%)
Others	767 (52.5%)
Educational level	
University or higher	966 (66.2%)
Others	494 (33.8%)
Working status	
Full-time	925 (63.4%)
Others	535 (36.6%)
Other variables	
Number of stressful concerns	3.2 (1.8)
Help-seeking intention	
Yes	665 (45.6%)
No	795 (54.4%)
Ways of coping	
Positive coping	852 (58.4%)
Others	608 (41.6%)
Study variables	
Caring role	
Yes	672 (46.0%)
No	788 (54.0%)
Caring for (multiple responses)	
Elderly	156 (10.7%)
Chronically ill	89 (6.1%)
Disable person	62 (4.2%)
Young children	321 (22.0%)
Others	237 (16.2%)
Insomnia	
Moderate to severe	279 (19.1%)
None to mild	1181 (80.9%)
Outcome variable	
Mental Well-being	
Overall	21.5 (4.9)
Male	21.2 (5.6)
Female	21.6 (4.6)

The bivariate relationships between the demographics, study variables, and the outcome were analysed, and the results are summarised in **Table 2**. All variables of interest were significantly associated with mental well-being, unadjusted for other variables. The demographic variables were then included in further analysis.

**Table 2:** Bivariate association between demographics and other variables, caring roles, insomnia, and mental well-being (N=1460).

Variables	Results of association
Demographics	
Sex	t_1458_ = -2.31, p=0.021
Age group	F_(2, 1457)_ = 82.25, p<0.001
Marital status	t_1458_ = -7.48, p<0.001
Educational level	t_1458_ = 2.05, p=0.041
Working status	t_1458_ = -2.44, p=0. 015
Number of stressful concerns	r = -0.28, p<0.001
Help-seeking intention	t_1458_ = 6.05, p<0.001
Ways of coping	t_1458_ = -8.52, p<0.001
Study variables	
Caring role	t_1458_ = -5.15, p<0.001
Insomnia	t_1458_ = 12.05, p<0.001

The association between caring roles and mental well-being was examined with adjustment for other variables. The results were tabulated in **Table 3**. As shown, the relationship remained significant after controlling for age group, insomnia, and other potential confounding variables with a regression coefficient of 0.65 (s.e. = 0.30) (t=2.18, p=0.029). To examine the moderating role of age group in the relationship between caring roles and mental well-being, the interactive terms of caring role and age group were tested. The results are also presented in **Table 3**. These results indicated that there was an overall significant interaction between caring roles and age group (β=0.84, s.e. = 0.10, t=8.42, p<0.001) and significant group differences were observed using the youngest non-carer as the reference group (**Table 3**). Hence, further analyses of the moderating role of insomnia in the association between caring roles and mental well-being were conducted for each age group.

**Table 3:** Results obtained from the multiple regression analyses on the interaction effect of caring role and age on mental well-being.

Variable of interest	Regression Coefficient	Standardise β	Standard Error	t-value	Significance p
Caring role^a^	0.65	0.06	0.30	2.18	0.029
Interaction terms between caring role (yes/no) and age groups^b^					
Caring role*age group	0.84	0.22	0.10	8.42	<0.001
No*18-34 (Reference)	-	-	-	-	-
No*35-54	2.32	0.14	0.45	5.18	<0.001
No*55+	4.00	0.17	0.63	6.31	<0.001
Yes*18-34	1.01	0.06	0.47	2.17	0.030
Yes*35-54	2.49	0.19	0.41	6.13	<0.001
Yes*55+	5.13	0.24	0.59	8.71	<0.001

a Adjusted for age group, insomnia, sex, marital status, education levels, employment status, number of stressful concerns, coping, and help-seeking.

b Adjusted for insomnia, sex, marital status, education levels, employment status, number of stressful concerns, coping, and help-seeking

The results of the sub-group analyses for the moderating role of insomnia in the association between the caring roles and mental well-being are presented in **Table 4**. After adjusting for potential confounders, the results suggested that there was an overall significant interaction between caring roles and insomnia for the 18-34 years (β=1.55, s.e. = 0.55, t=2.81, p=0.005), but not for other age groups (**Table 4**). Further analyses of comparing the mental well-being of different insomnia groups among carers for each age group indicated that the mental well-being scores of respondents with moderate to severe insomnia were significantly lower than that of the none to mild insomnia with an average of 3.9 units (s.e. = 0.73) (t= -5.43, p<0.001) in 35-54 years and 3.3 units (s.e. = 0.98) ((t= -3.36, p=0.001) among those 55 years or older. However, there was no statistically significant difference in the mental well-being scores between insomnia groups in the youngest age group (t= -0.84, p=0.401). For the non-carers, significant differences were found between insomnia groups across all age groups, with a reduction of the mean mental well-being scores ranging from 3.62 to 4.31 for moderate and severe insomnia in the oldest and the youngest age groups.

**Table 4:** Results obtained from the multiple regression analyses on the interaction effect of caring role and insomnia on mental well-being for each age group.

Interaction terms between caring role (yes.no) and insomnia^a^	Regression Coefficient	Standardise β	Standard Error	t-value	Significance p
18-34 years					
Caring role *insomnia	1.55	0.37	0.55	2.81	0.005
No*none/mild (Reference)	-	-	-	-	-
No*moderate/severe	-4.31	-0.28	0.57	-7.51	<0.001
Yes*none/mild	0.03	0.01	0.53	0.006	0.949
Yes*moderate/severe	-1.01	-0.04	0.85	-1.18	0.237
35-54 years					
Caring role *insomnia	-0.30	-0.08	0.60	-0.49	0.622
No*none/mild (Reference)	-	-	-	-	-
No*moderate/severe	-3.67	-.015	0.10	-3.68	<0.001
Yes*none/mild	0.36	0.03	0.51	0.70	0.482
Yes*moderate/severe	-3.69	-0.21	0.79	-4.69	<0.001
55 years or older					
Caring role *insomnia	-0.13	-0.04	0.88	-0.15	0.882
No*none/mild (Reference)	-	-	-	-	-
No*moderate/severe	-3.62	-0.20	1.28	-2.84	0.005
Yes*none/mild	1.27	0.12	0.82	1.54	0.125
Yes*moderate/severe	-1.70	-0.10	1.26	-1.35	0.179

a Adjusted for insomnia, sex, marital status, education levels, employment status, number of stressful concerns, coping, and help-seeking.

## Discussions

The aging population is a common global phenomenon that affects nearly every country. As part of the natural development accompanying such a phenomenon, the demands for informal and unpaid caregiving provided by family members or relatives for patients with disabilities and chronic diseases have grown in recent years. The burden of care is not only shouldered by adults but also by many young people. Previous studies have shown that, although having a caring role may have a negative effect on young caregivers, there are also positive effects on their mental well-being (17–23). Given the established fact that sleep quality, particularly insomnia, is a crucial risk factor for mental well-being, this motivates the current study to examine the possible moderating effect of age and insomnia in the relationship between caregiving and mental well-being. The results indicated that the mental well-being of the respondents, on the whole, is lower than the latest reported adult population norms in the UK of 23.7 for males and 23.2 for females (36). The study’s results further support the hypothesis that age and insomnia play a role in moderating the association between caregiving and mental well-being. These results suggest that, after controlling for the effect of confounding variables, there was no significant difference in mental well-being scores between insomnia groups among caregivers of 18-34 years. On the other hand, mental well-being was significantly reduced in the moderate to severe insomnia group among non-caregivers in comparison to those who had none to mild insomnia in the same age group. For other age groups, no such effect has been observed. Such results have not been reported previously in the literature, making comparison of results difficult. This also reflects that the findings of the current study are unique. It is also worth noting that the age and sex distributions of the sample are somewhat biased towards females and the younger age group. According to the population projection for 2024 published by the Statistics and Census Service of the Government of Macao SAR, the male and female distributions of the adult population were about 46% and 54%. For the age distributions by age groups, it was estimated that young adults aged under 35 years constituted 32% of the adult population, about 36% aged between 35 and 54 years, and 32% aged 55 years or older. Hence, the results might have reflected a gender-biased phenomenon (37).

The results obtained have both empirical and theoretical implications. On the empirical side, they support the notion that caregiving may have a positive effect on both the caregiver and the person being cared for, particularly for young caregivers. Previous studies have shown the association between caregiving and mental well-being with improving self-esteem, self-growth, connectivity with others, and self-identity in young caregivers (17–21, 32, 33). The results of no statistically significant difference in the mental well-being scores between insomnia groups in the caregivers of the youngest age group further suggest that the association between caregiving and mental well-being is not affected by the degree of insomnia. On the other hand, mental well-being has been affected by the degree of insomnia among young non-caregivers. These results could be interpreted as the detrimental effect of insomnia on mental well-being has been attenuated by caregiving since the relationship between the interaction of caring roles and insomnia and mental well-being is bidirectional. Following this interpretation, there could also be a potentially practical implication. Caregiving may play an important role in lightening the impact and burden of insomnia on the mental well-being of young people. In other words, active involvement in caring for others may benefit young people suffering from moderate to severe insomnia in enhancing their mental well-being.

As in all studies, strengths and weaknesses are identified in this study. For its strengths, the survey used validated and standardised instruments for assessing insomnia and mental well-being, designed with a solid theoretical basis. The use of well-validated instruments for data collection on the study and outcome variables helps to reduce the information basis that could be introduced to the study. Another strength is that the sample was generated with the involvement of a large number of different associations and organisations, including large NGOs, professional bodies, community groups, and charity organisations. These bodies represent a range of Macau residents in the community and different sectors of society. Additionally, using the online questionnaire with a platform that can cross-check the validity of information input by the respondents ensures the data quality. However, the shortcoming of the data collection mechanism is that it only provides data on the fully completed questionnaire. In that case, data from the partially completed questionnaires are lost, and comparisons between full and partial respondents cannot be conducted to examine any response bias. Another weakness of the study is the age and gender bias of the sample. The implications have been discussed above. A further weakness is that information obtained on the main study variable, namely the caregiving roles, was based on a single question, not a validated instrument. This could introduce information bias to the study. However, such bias is likely to the nondifferential rather than differential. For future studies, it is recommended to use a standardised and validated assessment instrument, such as the Informal Caregiver Burden Assessment Questionnaire (QASCI) (38).

In conclusion, the study’s results suggest that age and insomnia play a moderating role in the association between caregiving and mental well-being. These results further support the notion that being a caregiver may have some benefits for the mental well-being of younger caregivers but not for caregivers of older age. In any case, caregiving has an impact on the mental well-being of caregivers. With the growing magnitude of caregiving globally, this is a major public mental health issue needing more immediate attention.

## Data Availability

The datasets presented in this article are not readily available because The dataset was generated as a collaborative partnership between the Faculty of Medicine, Macau University of Science and Technology and the Mental Health Association Hong Kong and Macau. Release of data, apart from the results of data analyses, must have the permission from both bodies. Request of data could be considered by an application to the author for a joint decision of the partnership. Requests to access the datasets should be directed to Lawrence Lam, tmlam@must.edu.mo.
